# Supplementary material for: Development of the Conceptualization of Pain Questionnaire: A Measure to Study How Children Conceptualize Pain
Source: Int J Environ Res Public Health. 2021 Apr 6;18(7):3821. doi: 10.3390/ijerph18073821 (PMC8038728; doi:10.3390/ijerph18073821)
Supplement: Supplementary file 1 [file ijerph-18-03821-s001.pdf]

Table S1: Original Catalan version of the Conceptualization of Pain Questionnaire (COPAQ)

Afirmació	Ver	Fals	No ho sé
Només tinc dolor quan estic lesionat o estic a punt de lesionar-me.	O	O	O
Les persones que tenen dolor durant molt de temps, segur que tenen alguna cosa que no s'ha curat del tot.	O	O	O
Quan tinc dolor és perquè el meu cos li envia informació dolorosa al cervell.	O	O	O
Si un medicament no em treu el dolor, la lesió és més greu del que em semblava.	O	O	O
El meu cervell decideix quan haig de tenir dolor.	O	O	O
El dolor que sento depèn de la situació en la que em trobo.	O	O	O
Podria ser que tingués dolor i no me n'adonés.	O	O	O
Si em lesiono, segur que tindré dolor.	O	O	O
Si a algú se'l pot distreure del seu dolor això vol dir que el seu dolor no és real.	O	O	O
Una mateixa lesió produeix la mateixa intensitat de dolor en diferents persones.	O	O	O
Si un dolor varia d'intensitat segons l'estat d'ànim, aquest dolor no és real.	O	O	O
Tenir dolor durant molt de temps, implica que ja es tindrà dolor per sempre.	O	O	O
Puc tenir dolor encara que no tingui cap lesió.	O	O	O
De vegades, el dolor pot venir de pensar que t'has fet mal, encara que estiguis bé.	O	O	O
Una lesió més greu provocarà més dolor que una lesió menys greu.	O	O	O
